# Chemogenetic Modulation of Electroacupuncture Analgesia in a Mouse Intermittent Cold Stress-Induced Fibromyalgia Model by Activating Cerebellum Cannabinoid Receptor 1 Expression and Signaling

**DOI:** 10.3390/life15091458

**Published:** 2025-09-17

**Authors:** I-Han Hsiao, Ming-Chia Lin, Hsin-Cheng Hsu, Younbyoung Chae, Yi-Kai Su, Yi-Wen Lin

**Affiliations:** 1 School of Medicine, College of Medicine, China Medical University, Taichung 404328, Taiwan; 018309@tool.caaumed.org.tw; 2 Department of Neurosurgery, China Medical University Hospital, Taichung 404332, Taiwan; 3 Department of Nuclear Medicine, E-DA Hospital, I-Shou University, Kaohsiung 82445, Taiwan; ed101186@edah.org.tw; 4 Department of Traditional Chinese Medicine, China Medical University Hsinchu Hospital, Hsinchu 302233, Taiwan; 002762@tool.caaumed.org.tw; 5 Acupuncture and Meridian Science Research Center, Kyung Hee University, Seoul 02453, Republic of Korea; ybchae@khu.ac.kr; 6 Department of Anesthesiology, E-DA Hospital, I-Shou University, Kaohsiung 82445, Taiwan; 7 Graduate Institute of Acupuncture Science, College of Chinese Medicine, China Medical University, Taichung 404328, Taiwan; 8 Chinese Medicine Research Center, China Medical University, Taichung 404328, Taiwan

**Keywords:** chemogenetics, electroacupuncture, fibromyalgia, CB1, pERK

## Abstract

Fibromyalgia (FM) is characterized by widespread musculoskeletal pain and tenderness, cognitive dysfunction, fatigue, and insomnia. Electroacupuncture (EA) has documented efficacy against FM-associated pain, while cannabinoid receptor 1 (CB1) plays a critical role in endogenous analgesia. Herein, we examined whether pain relief initiated by EA was linked with differing cerebellar CB1 levels and signaling in an intermittent cold stress (ICS) mouse model of FM. FM-like hyperalgesia and recovery were assessed by measuring mechanical and thermal nociceptive thresholds. Compared to control mice, ICS-induced FM-model mice exhibited a significantly reduced mechanical withdrawal threshold (2.3 ± 0.1 g) and shorter thermal withdrawal latency (4.0 ± 0.5 s), indicative of mechanical and thermal hyperalgesia. Both conditions were reversed by 2 Hz EA but not sham EA. Hyperalgesia was associated with reduced CB1 receptor expression and the enhanced activity of multiple nociceptive signaling pathways (PKA, PI3K, Akt, mTOR, ERK, and NF-kB) in the mouse cerebellum. The 2 Hz EA treatment reliably reversed these abnormalities, while the sham EA treatment did not. Intracerebroventricular injection of the CB1 agonist anandamide (AEA) recapitulated the effects of EA on pain thresholds, while the analgesic effects of EA were blocked by the CB1 antagonist AM251. Precise chemogenetic stimulation at the paraventricular nucleus (PVN) of the hypothalamus reliably induced FM pain. Chemogenetic inhibition at the PVN diminished FM through the CB1 pathway in the cerebellum. Our findings suggest that dysregulation of CB1 expression and aberrant hyperactivity of nociceptive signaling pathways in the cerebellum contribute to the etiology of FM and that the upregulation of CB1 signaling mediates the analgesic efficacy of EA.

## 1. Introduction

Fibromyalgia (FM) is a chronic illness characterized by general, intense, and sustained pain, the cause of which remains unknown. The pain typically affects muscles, ligaments, tendons, and soft tissues, particularly in the shoulders, back, chest, arms, and legs. Symptoms often appear between the ages of 20 and 50 years and are exacerbated by cold weather, depression, anxiety, and inflammation [1,2]. Although several drugs have been approved by the United States Food and Drug Administration for symptom relief, such as duloxetine, pregabalin, and milnacipran, no curative treatments exist for FM. Current approaches to treatment involve multidisciplinary methodologies such as clinical pharmacological medication, non-invasive transcutaneous electrical nerve stimulation [3], and medical cannabis [4]. FM is highly comorbid with conditions such as depression, anxiety, rheumatoid arthritis, systemic lupus erythematosus, osteoarthritis, and other chronic pain disorders [5,6,7]. Löfgren et al. reported an increased functional connectivity between the caudate and cerebellum in FM patients; however, the cerebellum has received relatively little attention in pain-related fields. Accordingly, we aimed to elucidate the efficacy of electroacupuncture (EA) treatment and cellular mechanisms in the cerebellum in relieving FM pain [8].

While the precise cause of FM remains unclear, recent studies have highlighted abnormalities in brain, immune, and endocrine functions, including glial activation. Brain dysfunction in FM may involve deficits in endogenous analgesic systems, such as the endogenous cannabinoid (CB) system [9,10], which is crucial for maintaining human health. CBs were first isolated from cannabis leaves, and endocannabinoids have been found in various tissues throughout the body. These molecules bind to CB receptors 1 and 2 (CB1 and CB2). These receptors belong to the family of G-protein-coupled receptors with seven transmembrane domains that activate downstream signaling cascades via the Gi and Go proteins. The activation of CB1 receptors inhibits adenylate cyclase, leading to reduced intracellular cAMP and protein kinase A (PKA) activity. This pathway results in the inhibition of voltage-gated calcium channels and the activation of potassium channels [8,9,10], resulting in reduced neuronal excitability. Additionally, CB1 receptors modulate mitogen-activated protein kinases such as extracellular signal-regulated kinase (ERK), c-jun N-terminal kinase (JNK), and p38. Conversely, CB2 receptors primarily regulate immune signaling and may influence neuroinflammation through microglial cells [9,10,11].

Acupuncture needling involves inserting very fine steel needles into the skin or muscles at various acupoints to treat specific afflictions, with pain relief being one of the most common indications. For instance, acupuncture is used to relieve chemo-therapy-induced pain, dental pain, labor pain, lower back pain, and FM, among other conditions [12,13,14]. We recently reported that acupuncture in mice can increase the concentrations of adenosine triphosphate, interleukin-1β, interleukin-6, glutamate, substance P, and histamine in the regional microenvironments of acupoints [15]. These potential therapeutic effects may be enhanced by concomitant electrical stimulation, or EA. Lin and colleagues (2020) reported that EA treatment attenuated FM pain and that this analgesic effect was associated with reduced plasma concentrations of inflammatory cytokines, including interleukins, tumor necrosis factor-α, and interferon-γ [16]. We further demonstrated that EA reduced pain levels in a mouse model of inflammatory pain, possibly by affecting adenosine and opioid receptor signaling [17,18]; it has also been shown to decrease inflammatory, neuropathic, and FM-related pain in numerous models [19,20,21,22,23,24,25,26]. Acupuncture has also been used to effectively alleviate obesity-induced depression by regulating inflammatory cytokines and the transient receptor potential vanilloid member 1 pathway [27].

This research is indicative of the noteworthy role of CB1 in the cerebellum of FM mice and serves to support the rationale behind its investigation in the current study. We report that 2 Hz EA, but not sham EA, attenuated mechanical and thermal hyperalgesia in FM-model mice and reversed both CB1 receptor downregulation and pain-related signaling factor hyperactivity in the cerebellum of FM mice. Intracerebroventricular (i.c.v.) injection of the CB1 agonist anandamide (AEA) replicated the effects of EA, while i.c.v. injection of a CB1 antagonist significantly diminished EA-induced analgesia. Chemogenetic activation at the paraventricular nucleus (PVN) reliably replicated FM pain, while chemogenetic inhibition at the PVN reduced FM pain through the CB1 pathway in the mouse cerebellum. This study supports the efficacy of EA for managing FM pain and highlights the CB1 receptor as a major therapeutic target.

## 2. Materials and Methods

### 2.1. Experimental Animals

The investigational rodents used in this study were 8–12 weeks old female C57B/L6 mice (weighing about 18–20 g). Female mice were used in this experiment to reflect the clinical reality that the prevalence of FM in women is higher than that in men. We purchased experimental animals from Taiwan Biolasco (Yilan, Taiwan). After the arrival of the animals, we placed the mice in animal cages and maintained an environment of 25 °C, 60% humidity, and a 12 h light/dark cycle. The proposal was reviewed by the Animal Care and Use Committee of China Medical University (license number: CMUI-ACUC-2021-336) and followed the Guide for the Use of Laboratory Animals of the National Academy of Sciences Press. The investigators were blinded with regard to group division and data examination. We used the G*Power 3.1.9.7 program to calculate the required sample size. The results showed that at least 9 mice per group was the minimum number of experiments required to achieve a significant alpha level of 0.05 and a power of 80%. The mice were randomly divided into the following four groups: (1) normal mice (normal group); (2) cold stress-induced FM pain (FM group); (3) FM pain treated with 2 Hz EA (FM + 2 Hz EA); and (4) FM pain treated with sham EA (FM + sham EA).

### 2.2. Modeling Fibromyalgia

We placed animals in a 4 °C environment to establish the mouse FM model and kept the normal mice at room temperature. At 10 a.m. the next day, we returned the FM mice to room temperature for 30 min and then to 4 °C for 30 min. This action was repeated until 4 p.m., and the animals were returned to 4 °C until the third day [28,29].

### 2.3. Electroacupuncture

Animals were first sedated using sequential administration of 5% isoflurane for induction and 1% for maintenance. Two 1-inch-long stainless steel acupuncture needles (32G, Taiwan Yuguang Co., Ltd., Yuyao, China) were inserted into the bilateral ST36 acupoints [16,17]. Electrical stimulation was delivered through 100 μs square pulses at 2 Hz with an intensity of 1 mA using a Trio 300 (Ito, Japan). EA was performed on days 3 and 4 following FM model induction. The sham EA group was treated with the same procedure as the EA group but without an electric current.

### 2.4. Mechanical and Thermal Nociception Tests

Mice were placed in separate Plexiglass boxes perforated on the top face, placed on an elevated horizontal wire mesh stand, covered with a dark cloth, maintained in a silent environment at room temperature (25 °C), and allowed to habituate for 30 min before starting the behavioral test. The experiments were only conducted when the mice were calm, all their feet were placed on the surface, and not grooming or sleeping. The von Frey filament measuring instrument (IITC Life Science Inc., Woodland Hills, CA, USA) was used to increase pain pressure in the center of the right plantar hind paws of the mice. The maximum pressure was achieved when the right hind paw was lifted using the plastic tip and the mouse reflexively withdrew its hind paw. We allowed for 3 min breaks between stimuli. The results were recorded as mechanical sensitivity. Hargreaves’ test was used for measuring thermal sensitivity, with preparations similar to the von Frey test. The subjects were placed in an animal enclosure that separated the mice to limit interaction and covered using a dark cloth. After allowing for habituation, the experiments were initiated. An IITC Plantar Analgesia Meter (IITC Life, Sciences, SERIES8, Model 390G) was used to measure the withdrawal latency time of mice subjected to radiant heat applied on the surface, targeting the center of the hind paw. The heat source stimulation did not exceed 20 s to avoid paw injury. The pain threshold was calculated based on the time of withdrawal after stimulation.

### 2.5. Western Blotting

Mice cerebella were excised, put on ice, and kept at −80 °C till extraction. Retrieved tissues were homogenized in cold radioimmunoprecipitation buffer containing 5 mM EDTA, 50 mM Tris-HCl, 1% NP-40, pH 7.4, 250 mM NaCl, 50 mM NaF, 1 mM Na_3_VO_4_, 0.02% NaN3, and 1× protease inhibitor cocktail (AMRESCO). Total contains were separated by 8% SDS gel electrophoresis and shifted to PVDF membranes for Western blotting. Membranes were incubated with 5% non-fat milk in Tris buffer with Tween 20 (TBS-T, 10 mM Tris pH 7.5, 100 mM NaCl, 0.1% Tween 20), then rinsed with the first antibody against anti-tubulin (1:5000, Merck, Rahway, NJ, USA), CB1 (1:1000, Alomone, Jerusalem, Israel), pPKA (1: 1000, Alomone, Jerusalem, Israel), pPI3K (1:1000, Millipore, Burlington, MA, USA), pPKC (1:1000, Millipore, MA, USA), pAkt (1:1000, Millipore, MA, USA), pmTOR (1:500, Millipore, MA, USA), pERK (1:1000, Millipore, MA, USA), and pNFκB (1:1000, Millipore, MA, USA) for 1 h at room temperature, followed by incubation with peroxidase-conjugated anti-rabbit or anti-mouse (1:5000). Target protein outcomes were imagined via an enhanced chemiluminescent substrate kit (PIERCE) and captured on a LAS-3000 Fujifilm imaging system (Fuji Photo Film Co., Ltd., Tokyo, Japan). The concentrations of proteins were calculated using NIH Image J 14.7 J (Bethesda, Rockville, MD, USA). α-tubulin was used as an internal control.

### 2.6. Immunofluorescence

Animals were sacrificed using 5% isoflurane and intracardially irrigated with normal saline mixed with 4% paraformaldehyde. Samples were submerged in 30% sucrose at 4 °C overnight for cryoprotection and implanted in optimal-cutting-temperature composite. Frozen tissues were chopped into 20 μm pieces with a cryostat. Sections were mixed with 4% paraformaldehyde and nurtured with 3% BSA, 0.1% Triton X-100, and 0.02% sodium azide for over 1 h. Sections were then incubated overnight in saline with 1% BSA containing first antibodies (1:200, Alomone) against the CB1 receptor and pERK. Tissue sections were then incubated with Alexa 488-conjugated IgG (H + L) and Alexa 594-conjugated IgG (H + L) for 2 h at room temperature, mounted with cover slips, and examined using fluorescence microscopy.

### 2.7. CB1 Receptor Agonist and Antagonist Administration

The effects of CB1 agonist and antagonist injections on nociception were examined in adult C57BL/6 female mice (*n* = 6 per group). The specific CB1 agonist AEA (Sigma, St. Louis, MO, USA) was administered with ICV injection at 100 μM, while the CB1 antagonist AM251 (Sigma, St. Louis, MO, USA) was administered using ICV injection at 5 µg per mouse in 10 µL of saline under light isoflurane anesthesia (1%).

### 2.8. Chemogenetic Operation

Animals were sedated with 0.5–1% isoflurane with the head kept immobile in a stereotaxic apparatus. A cannula was implanted at the PVN, 0.82 mm posterior and 0.2 mm lateral of bregma at 0.8 mm below the skull, and then stabilized with dental glue. A total of 0.3 μL of virus-related liquid was then injected for an extra 3 min with a syringe pump (KD Scientific, Holliston, MA, USA). The inoculation duct was kept in the PVN for an additional 2 min to allow the liquid to emit. A hM3Dq or hM4Di DREADD (designer receptor exclusively activated by designer drugs: AAV8-hSyn-hM4D(Gi)-mCherry or AAV8-hSyn-hM4D(Gi)-mCherry; Addgene Plasmid #50477 and #50475, MA, USA) was inoculated at the PVN. Clozapine N-oxide (CNO; Sigma C0832, St. Louis, MO, USA) was used to activate the DREADD. CNO was dissolved in 5% dimethyl sulfoxide (DMSO; Sigma D2650) before 1 mg/kg intraperitoneal inoculation on day 4.

### 2.9. Statistical Analyses

All statistical analyses were conducted with SPSS 21.0 software. The results are expressed as the mean ± standard error. Group means for each day were compared using one-way analysis of variance, followed by Tukey’s tests for pairwise comparisons. A *p* value of <0.05 was considered statistically significant for all tests.

## 3. Results

### 3.1. Attenuation of Intermittent Cold Stress-Induced Fibromyalgia-like Pain by Electroacupuncture

**Figure 1:**
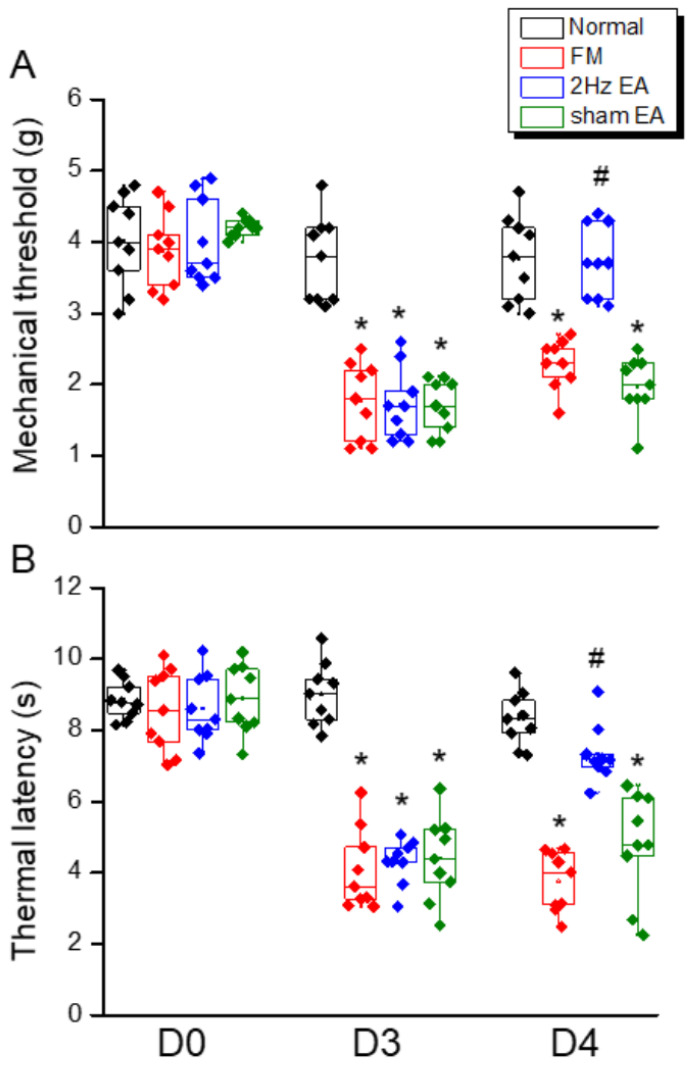
Mechanical and thermal hyperalgesia (elevated pain thresholds) in a mouse model of fibromyalgia (FM) induced by intermittent cold stress (ICS) was reversed by electroacupuncture (EA). (**A**) Mechanical pain threshold in normal control, FM, FM with 2 Hz EA (2 Hz EA), and FM with sham EA (sham EA) groups as measured by von Frey filament tests. (**B**) Thermal pain thresholds in the same groups as measured using Hargreaves’ test for thermal withdrawal latency. Pain thresholds were reduced by ICS, and this hyperalgesia was completely reversed by 2 Hz FM but not sham FM. * *p* < 0.05 compared to the normal mice. ^#^ *p* < 0.05 compared to the FM mice. *n* = 9.

We first examined whether 2 Hz EA could significantly attenuate mechanical and thermal hyperalgesia in FM-model mice during the initial transition from acute to chronic pain (the subacute phase). To confirm successful induction of the FM model, we compared von Frey hair responses and Hargraves thermal withdrawal responses between the ICS-exposed and ICS-unexposed mice. Both mechanical pain thresholds (Figure 1A, red column, day 4: 2.3 ± 0.1 g, *n* = 9) and thermal pain withdrawal latencies (Figure 1B, red column, day 4: 4.0 ± 0.5 s, *n* = 9) were significantly lower in model mice. Bilateral 2 Hz EA reliably increased mechanical pain thresholds (reduced mechanical hyperalgesia: Figure 1A, blue column, day 4: 3.7 ± 0.2 g, *n* = 9) and prolonged thermal latency (reduced thermal hyperalgesia: Figure 1B, blue column, day 4: 7.3 ± 0.3 s, *n* = 9), while sham EA had no effect, consistent with our previous studies.

### 3.2. Reduced Expression of CB1 and Enhanced Activation of Pain-Related Signaling Factors in the Cerebellar C5 Region of FM-Model Mice Were Reversed by 2 Hz EA

**Figure 2:**
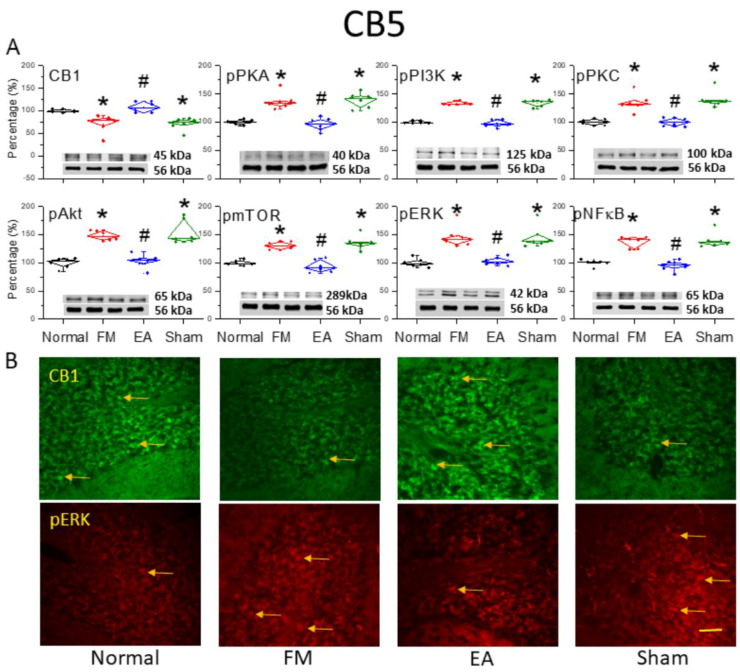
Downregulation of the cannabinoid 1 (CB1) receptor and upregulation of pain-associated signaling factors by ICS and reversal by 2 Hz EA in the cerebellar CB5 region. (**A**) Densitometric analysis of target protein bands (CB1, pPKA, pPI3K, pPKC, pAkt, pmTOR, pERK, and pNF-κB) on Western blots of cerebellar tissues from normal, FM, FM + EA, and FM + sham EA group mice. * *p* < 0.05 vs. the normal mice; ^#^ *p* < 0.05 vs. the FM group. Mean ± SEM of *n* = 6 mice per group. α-tubulin (56 kDa) was used as an internal control (lower chart of raw Western blot band). (**B**) Immunohistochemical staining of cerebellar CB5 slices from the same groups showing CB1 (green) and pERK (red) immunofluorescence. Yellow arrows indicate immune-positive signals. Bar = 100 μm. Typical results for *n* = 3 mice per group.

Western blotting of cerebellar lysates harvested from the four treatment groups (normal, FM, FM + 2 Hz EA, FM + sham EA) revealed reduced expression of CB1 receptors (Figure 2A, * *p* < 0.05, *n* = 6) and increased expression of phosphorylated PKA (pPKA) in the CB5 region of FM-model mice (Figure 2A, * *p* < 0.05, *n* = 6) compared to normal controls. However, 2 Hz EA but not sham EA dramatically reversed these expression changes (Figure 2A, ^#^ *p* < 0.05, *n* = 6). The expression of phosphorylated phosphoinositide 3-kinase (pPI3K) and pPKC were elevated in the CB5 region of FM mice (Figure 2A, * *p* < 0.05, *n* = 6), and were reversed by 2 Hz EA (Figure 2A, ^#^ *p* < 0.05, *n* = 6). Phosphorylation levels of the downstream phosphorylated protein kinase B (pAkt) and mammalian target of rapamycin (pmTOR) were also higher in the FM group compared to the normal group (Figure 2A, * *p* < 0.05, *n* = 6), and 2 Hz EA but not sham EA reliably downregulated pAkt and pmTOR expression (Figure 2A, ^#^ *p* < 0.05, *n* = 6). Additionally, pERK expression was augmented in the CB5 region of FM mice (Figure 2A, * *p* < 0.05, *n* = 6) and was reversed by 2 Hz EA (Figure 2A, ^#^ *p* < 0.05, *n* = 6). The transcription factor phosphorylated nuclear factor kappa-light-chain-enhancer of activated B cells (pNF-κB), a major mediator of stress responses, was also upregulated in FM mice and downregulated by 2 Hz EA (Figure 2A, *n* = 6). These results suggest that the observed antinociceptive effects of 2 Hz EA are mediated by reduced ICS-induced kinase–pNF-κB signaling and concomitant suppression of target genes involved in hyperalgesia. Western blot results were further supported by the immunohistochemistry of cerebellar tissues harvested from the four treatment groups (Figure 2B). ICS-induced FM reduced CB1 receptor immunoexpression in CB5, while 2 Hz EA but not sham EA reversed this effect on CB1 receptor expression. Similarly, increased pERK immunoexpression in the FM group was reversed by 2 Hz EA.

### 3.3. Reversal of CB1 Receptor Downregulation and ERK Upregulation in the Cerebellar CB6 Region by 2 Hz EA

**Figure 3:**
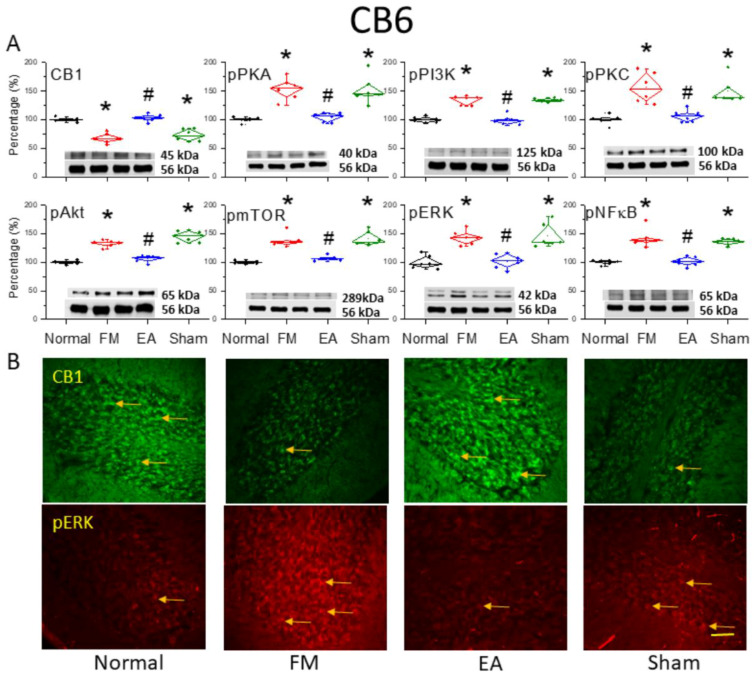
Downregulation of the cannabinoid 1 (CB1) receptor and upregulation of pain-associated signaling factors by ICS and reversal by 2 Hz EA in the cerebellar CB6 region. (**A**) Densitometry analysis of target protein bands (CB1, pPKA, pPI3K, pPKC, pAkt, pmTOR, pERK, and pNF-κB). * *p* < 0.05 vs. normal individuals. ^#^ *p* < 0.05 vs. the FM group. Mean ± SEM of *n* = 6 mice per group. α-tubulin (56 kDa) was used as an internal control (lower chart of raw Western blot band). (**B**) Immunofluorescence intensities of CB1 (green) and pERK (red) in the mouse CB6 region. Yellow arrows indicate immune-positive signals. Bar = 100 μm. Typical results for *n* = 3 mice per group.

We also examined whether 2 Hz EA reversed CB1 receptor downregulation in the CB6 region, a crucial region mediating FM-associated pain. After 4 days of ICS, CB6 tissues were collected and processed for Western blotting of CB1 and related kinases. Consistent with the CB5 results, CB1 receptor expression was downregulated in CB6 after ICS (Figure 3A, * *p* < 0.05, *n* = 6), while 2 Hz EA for 2 continuous days significantly reversed this effect (Figure 3A, ^#^ *p* < 0.05, *n* = 6). Amplified pPKA, pPI3K, and pPKC were also found in the CB6 region of the FM model (Figure 3A, * *p* < 0.05, *n* = 6) compared to normal controls. However, 2 Hz EA but not sham EA intensely reversed these expression changes (Figure 3A, ^#^ *p* < 0.05, *n* = 6). Similarly, pAkt and pmTOR were greater in the FM group (Figure 3A, * *p* < 0.05, *n* = 6), and 2 Hz EA but not sham EA consistently attenuated pAkt and pmTOR expression (Figure 3A, ^#^ *p* < 0.05, *n* = 6). In addition, pERK was meaningfully augmented in the CB6 region of FM mice (Figure 3A, * *p* < 0.05, *n* = 6) and was diminished by 2 Hz EA (Figure 3A, ^#^ *p* < 0.05, *n* = 6). The transcription factor pNF-κB was also increased in FM mice and alleviated by 2 Hz EA (Figure 3A, *n* = 6). Immunohistochemical staining confirmed reduced CB1 receptor labeling in the FM and FM + sham EA groups compared to controls, while CB1 receptor immunoexpression levels were substantially higher in the FM + 2 Hz EA group. Similar results were observed for pERK expression, which was reduced in the FM and FM + sham EA groups but reversed by 2 Hz EA (Figure 3B).

### 3.4. Reversal of CB1 Receptor Downregulation and Pain-Associated Kinase Upregulation by 2 Hz EA in the Cerebellar CB7 Region

**Figure 4:**
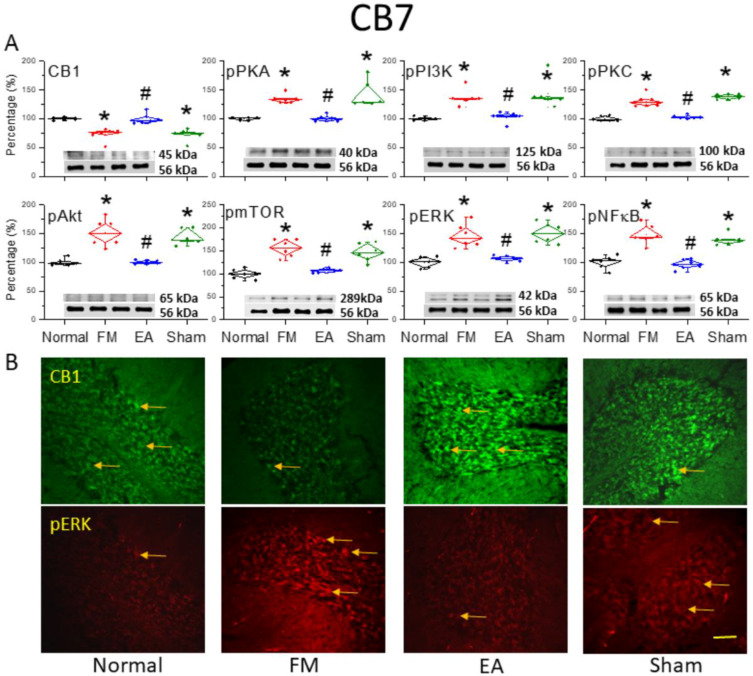
Downregulation of the cannabinoid 1 (CB1) receptor and upregulation of pain-associated signaling factors by ICS and reversal by 2 Hz EA in the cerebellar CB7 region. (**A**) Densitometric analysis of target protein bands (CB1, pPKA, pPI3K, pPKC, pAkt, pmTOR, pERK, and pNF-κB). * *p* < 0.05 vs. the normal group. ^#^ *p* < 0.05 vs. the FM group. *n* = 6. α-tubulin (56 kDa) was used as an internal control (lower chart of raw Western blot band). (**B**) Immunofluorescence intensities of CB1 (green) and pERK (red) in the mouse CB7 region. Yellow arrows indicate immune-positive signals. Bar = 100 μm. Typical results from *n* = 3.

We dissected the CB7 region to assess protein levels using Western blotting and immunofluorescence (Figure 4). ICS induction significantly decreased CB1 receptor expression in CB7 on day 4 (Figure 4A, * *p* < 0.05, *n* = 6). However, 2 Hz EA, but not sham EA, augmented CB1 receptor expression in ICS-exposed mice (Figure 4A, ^#^ *p* < 0.05, *n* = 6). In addition, pPKA, pPI3K, and pPKC levels were consistently elevated in ICS-exposed mice (Figure 4A, * *p* < 0.05, *n* = 6), and these effects were diminished by 2 Hz EA (Figure 4A, ^#^ *p* < 0.05, *n* = 6). Similarly to the aforementioned areas, the expression levels of pAkt, pmTOR, and pERK were significantly greater in ICS-initiated FM mice than in control mice (Figure 4A, * *p* < 0.05, *n* = 6), and these effects were reversed by 2 Hz EA (Figure 4A, ^#^ *p* < 0.05, *n* = 6). Induction of the FM-like condition also increased pNF-κB expression in CB7, consistent with the critical role of this area in FM pain development (Figure 4A, * *p* < 0.05, *n* = 6). This effect was abrogated by 2 Hz EA (Figure 4A, ^#^ *p* < 0.05, *n* = 6). In agreement with the Western blotting results, immunofluorescence staining (Figure 4B) revealed reduced CB1 immunoexpression and amplified pERK immunoexpression in the FM and FM + sham EA groups compared to controls, with 2 Hz EA reversing these effects (Figure 4B).

### 3.5. ICV Injection of a CB1 Agonist Reversed ICS-Induced Hyperalgesia While a CB1 Antagonist Blocked the Analgesic Effect of 2 Hz EA

**Figure 5:**
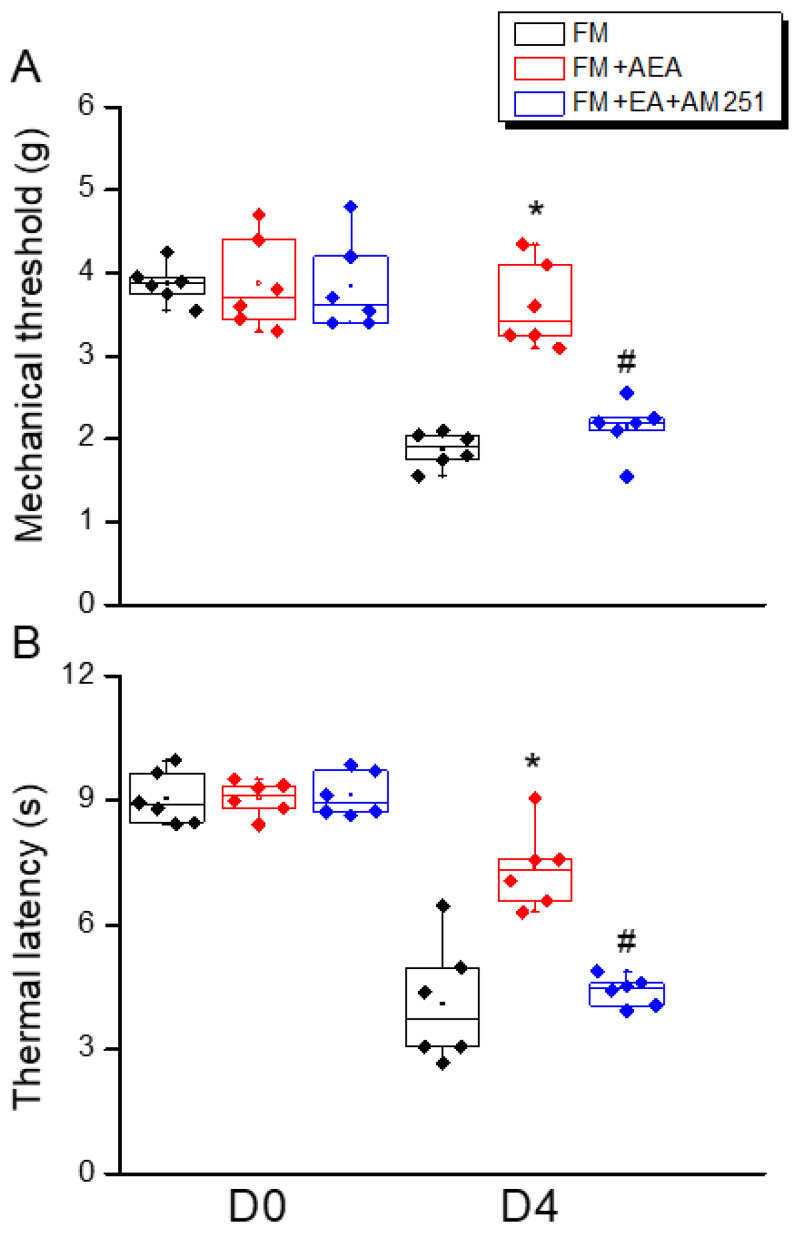
Recapitulation of EA-induced analgesia by intracerebroventricular injection of a CB1 agonist and blockade of EA-induced analgesia by a CB1 antagonist in FM-model mice. (**A**) Mechanical thresholds and (**B**) thermal latency thresholds of FM-group mice (black bars), FM mice treated with the CB1 agonist AEA (red bars), and FM mice receiving EA with the CB1 antagonist AM251 (blue bars). * *p* < 0.05 compared to the normal group. ^#^ *p* < 0.05 compared to the FM group. *n* = 9.

Before ICS induction, there were no substantial differences in responses to mechanical or thermal stimulation. In ICS-treated FM mice, mechanical and thermal hypersensitivity were observed, as previously described. Both mechanical (Figure 5A, red column, day 4: 3.6 ± 0.2 g, *n* = 9) and thermal hyperalgesia (Figure 5B, red column, day 4: 7.4 ± 0.4 s, *n* = 9) were significantly diminished by i.c.v. injection of the CB1 agonist AEA, indicating that CB1 receptor stimulation alone can recapitulate the painkilling effects of 2 Hz EA. Moreover, the pain-relieving effect of 2 Hz EA was negated by i.c.v. injection of the CB1 antagonist AM251 (Figure 5A,B, blue column, day 4: mechanical, 2.1 ± 0.1 g, and thermal, 4.4 ± 0.1 s, *n* = 9). Collectively, these results strongly suggest that the antinociceptive properties of 2 Hz EA treatment are intermediated by the activation of central CB1 receptors.

### 3.6. ICV Injection of a CB1 Agonist Reversed ICS-Induced Downregulation of CB1 Receptor and Upregulation of Nociceptive Signaling Factors in All Three Cerebellar Regions

**Figure 6:**
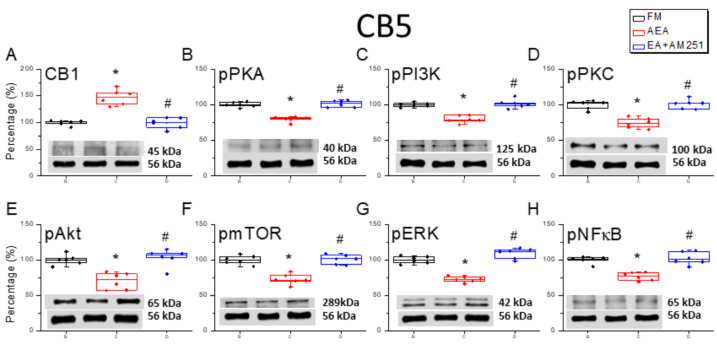
Effects of the CB1 agonist and antagonist on expression levels of CB1 and associated signaling factors in the cerebellar CB5 region. Expression levels were compared among the FM, FM + AEA, and FM + EA + AM251 groups using Western blots. (**A**–**H**) Densitometric analyses of (**A**) CB1, (**B**) pPKA, (**C**) pPI3K, (**D**) pPKC, (**E**) pAkt, (**F**) pmTOR, (**G**) pERK, and (**H**) pNF-kB expression. α-tubulin (56 kDa) was used as an internal control (lower chart of raw Western blot band). * *p* < 0.05 vs. the FM group. ^#^ *p* < 0.05 vs. the FM + AEA group. *n* = 6 per group.

**Figure 7:**
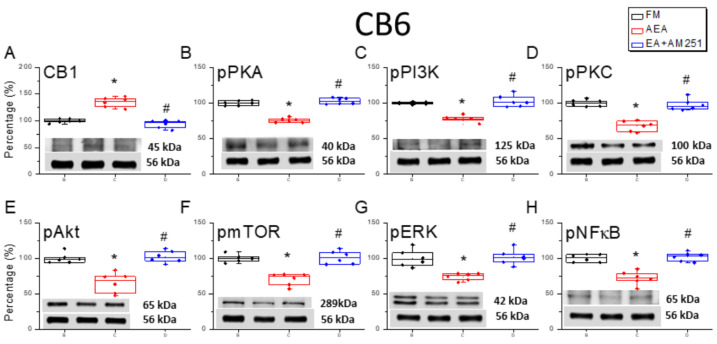
Effects of the CB1 agonist and antagonist on the expression levels of CB1 and associated signaling factors in the cerebellar CB6 region. Expression levels were compared among FM, FM + AEA, and FM + EA + AM251 groups using Western blots. (**A**–**H**) Densitometric analyses of (**A**) CB1, (**B**) pPKA, (**C**) pPI3K, (**D**) pPKC, (**E**) pAkt, (**F**) pmTOR, (**G**) pERK, and (**H**) pNF-kB expression. α-tubulin (56 kDa) was used as an internal control (lower chart of raw Western blot band). * *p* < 0.05 vs. the FM group, and ^#^ *p* < 0.05 vs. the FM + AEA group. *n* = 6 per group.

**Figure 8:**
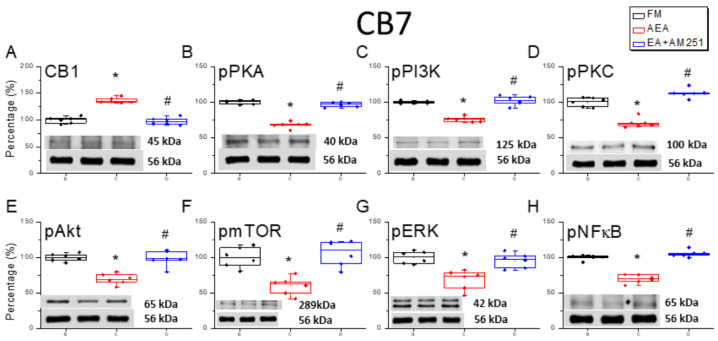
Effects of the CB1 agonist and antagonist on the expression levels of CB1 and associated signaling factors in the cerebellar CB7 region. Expression levels were compared among FM, FM + AEA, and FM + EA + AM251 groups using Western blots. (**A**–**H**) Densitometric analyses of (**A**) CB1, (**B**) pPKA, (**C**) pPI3K, (**D**) pPKC, (**E**) pAkt, (**F**) pmTOR, (**G**) pERK, and (**H**) pNF-kB expression. α-tubulin (56 kDa) was used as an internal control (lower chart of raw Western blot band). * *p* < 0.05 vs. the FM group. ^#^ *p* < 0.05 vs. the FM + AEA group. *n* = 6 per group.

Following our assessment of the effects of the CB1 agonist and 2 Hz EA on ICS-induced hyperalgesia, we performed Western blotting to examine the corresponding changes in CB1 receptor and nociceptive signaling molecule expression levels in the cerebellar regions CB5, CB6, and CB7. Surprisingly, downregulation of the CB1 receptor following FM modeling was reversed by ICV injection of the agonist AEA in CB5, CB6, and CB7 (Figure 6A, Figure 7A and Figure 8A, * *p* < 0.05, *n* = 6). Conversely, ICV injection of the antagonist AM251 reversed the upregulation of the CB1 receptor. Additionally, AEA injection reversed ICS-induced upregulation of pPKA, pPI3K, and pPKC (Figure 6B–D, Figure 7B–D and Figure 8B–D, * *p* < 0.05, *n* = 6), while ICV injection of the CB1 antagonist AM251 reversed the analgesic effects of 2 Hz EA (Figure 6B–D, Figure 7B–D and Figure 8B–D, ^#^ *p* < 0.05, *n* = 6). Qualitatively similar consequences were detected for the downstream nociceptive signaling factors pAkt, pmTOR, pERK, and pNF-κB. Expression levels were downregulated in all cerebellar regions subjected to ICV injection of AEA (Figure 6E–H, Figure 7E–H and Figure 8E–H, *n* = 6).

### 3.7. Chemogenetic Stimulation at Paraventricular Nucleus Induced Fibromyalgia and ICS-Induced Fibromyalgia Was Further Diminished by Chemogenetics

**Figure 9:**
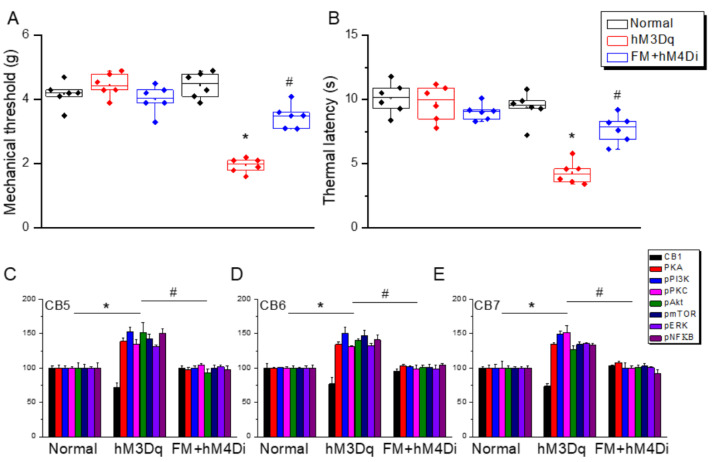
Chemogenetic tone on mice fibromyalgia pain through CB1 and related kinases. (**A**) Mechanical thresholds after CNO injection. (**B**) Thermal latency after CNO injection. (**C**) CB1 receptors and associated factors in CB5 regions of the mice receiving CNO stimulation. (**D**) CB1 and associated mediators in CB6 regions of the mice receiving CNO stimulation. (**E**) CB1 and associated molecules in CB7 regions of the mice receiving CNO stimulation. * *p* < 0.05 vs. normal rodents. ^#^ *p* < 0.05 vs. the hM3Dq individuals. *n* = 9 per group.

The paraventricular nucleus is known to be involved in pain signaling, especially in pain sensations in the cerebellum. We found that CNO activation of the PVN significantly initiated a chemogenetic-induced mechanical hypersensitivity (Figure 9A, hM3Dq, *n* = 9). Comparable consequences were perceived for thermal hyperalgesia at day 4 (Figure 9B, hM3Dq, *n* = 9). Chemogenetic downregulation of the PVN profoundly reduced mechanical and thermal pain (Figure 9A,B, FM + hM4Di, *n* = 9). Furthermore, we explored alterations in the CB1 pathway and proteins in regions of the mouse cerebellum. We found that CB1 was attenuated by chemogenetic stimulation of the PVN. The intensities of pPKA, pPI3K, and pPKC were all increased after CNO activation (Figure 9C–E, hM3Dq, *n* = 9). Similar consequences were also observed for pAkt, pmTOR, pERK, and pNFκB. Furthermore, chemogenetic inhibition of the PVN in FM mice significantly increased the expression of the CB1 pathway in the CB areas of FM mice. The aforementioned pain-related kinases were all diminished in CB regions after chemogenetic inhibition of the PVN (Figure 9C–E, FM + hM4Di, *n* = 9).

## 4. Discussion

A recent clinical trial revealed the efficiency of repetitive transcranial magnetic stimulation (rTMS) and transcranial direct current stimulation (tDCS) over the left dorsolateral prefrontal cortex (DLPFC) for reducing FM pain and improving patients’ quality of life. Specifically, two-thirds of FM patients in the rTMS group reported at least a 30% reduction in their visual analog scale (VAS) pain scores, and the entire rTMS treatment group demonstrated a significant time × group interaction in VAS scores from baseline to follow-up. These effects were observed after only three sessions of rTMS over the DLPFC [30]. Thus, various forms of electrical stimulation appear to reduce subjective FM-associated pain. Wang et al. reported that cannabis-based medicinal products improved sleep, anxiety, and quality of life in 306 FM patients from the UK medical cannabis registry [31]. Although the precise etiology of FM remains unclear, traumatic experiences may be a common precipitating factor. Mir’o et al. reported a higher incidence of traumatic experiences in FM patients, along with more PTSD-like symptoms compared to healthy controls, including insomnia, anxiety, depression, and functional impairment [32].

Recently, Mazza et al. reported that cannabis improved FM symptoms in patients resistant to current pharmacological therapies, as measured by a numerical rating scale, the Oswestry Disability Index, Hospital Anxiety and Depression Scale, widespread pain index, severity score, and side effect evaluation [33]. This further supports the potential of targeting CB1 receptors in FM therapy. Similarly, Sagy et al. found that medical cannabis was safe and effective for the treatment of FM symptoms, with side effects including dizziness, dry mouth, and gastrointestinal symptoms [4]. A recent study also reported that the myofascial technique reduced pain, central sensitization, and negative emotional symptoms while improving sleep quality among FM patients [34]. Similarly, Martínez-Pozas et al. observed reduced mechanical hyperalgesia in patients with chronic musculoskeletal pain following orthopedic manual therapy [35]. Albrecht et al. provided compelling in vivo evidence that microglial activation in the anterior and posterior middle cingulate cortices contributes to the onset of FM, as indicated by upregulation of the glial activation marker translocator protein (TSPO) [5].

Kawamura et al. combined electrophysiological and immunofluorescence analyses to demonstrate that CB1 is the main cannabinoid receptor at presynaptic synapses in the mouse cerebellum. Moreover, CB-dependent inhibition of excitatory transmission at cerebellar Purkinje cells was abolished in *Cb1^−/−^* mice, which exhibited very low CB1 expression as confirmed by immunochemical staining [36]. Consistent with the current findings, CB1 receptor agonists and inhibitors of endocannabinoid catalytic enzymes have yielded consistent analgesic effects in inflammatory and neuropathic pain models, although FM mouse models were not specifically examined. Nonetheless, such compounds have demonstrated efficacy in alleviating cancer, inflammatory, and neuropathic pain [37], strongly suggesting their therapeutic potential in FM management. Fanton et al. reported a positive relationship between anti-satellite glia cell IgG expression and FM severity. In addition, FM patients with high serum anti-SGC IgG concentrations exhibited pain-associated glial activation in the thalamus and rostral anterior cingulate cortex. Taken together, these results suggest that anti-SGC IgG levels are a useful diagnostic and prognostic tool for FM, potentially leading to novel antibody-based treatments [38].

## 5. Conclusions

**Figure 10:**
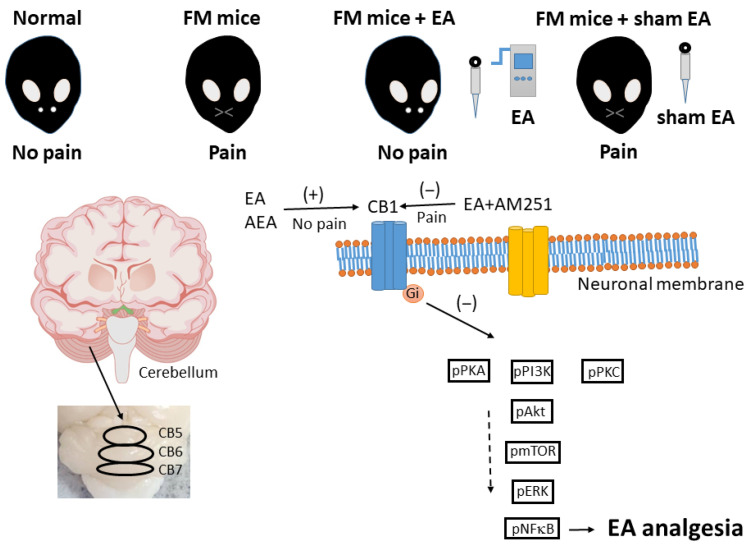
Graphic illustration of EA in FM treatment via CB1 and related molecules.

In conclusion, the current study demonstrates that ICS can induce FM-like mechanical and thermal hyperalgesia in mice by suppressing CB1 receptor signaling and promoting nociceptive signaling in mice cerebellum regions 5–7. Furthermore, both the hyperalgesic and molecular effects of ICS were reversed by 2 Hz EA (Figure 10).

### 5.1. Main Findings and Their Implications

Our main findings were that FM pain was associated with reduced CB1 receptor expression, accompanied by enhanced nociceptive kinases in the mice cerebellum. EA significantly reduced FM pain through augmenting CB1 receptors. Intracerebroventricular injection of the CB1 agonist or antagonist implied the precise mechanisms of CB1 receptor with regard to the analgesic effects of EA.

### 5.2. Study Strengths and Limitations

The accurate chemogenetic activation of the PVN consistently induced FM pain. By contrast, chemogenetic inhibition at the PVN diminished FM pain via the CB1 pathway. These data suggest that CB1 receptors in the cerebellum contribute to FM pain and the analgesic effect of EA. A limitation of the current study was that we only analyzed CB1 in this model and therefore cannot exclude additional receptors.

### 5.3. Future Perspectives

We suggest that future research investigates the use of EA at the ST36 acupoint with 150 μs square pulses at 2 Hz with an intensity of 1 mA. Postmortem scientific examinations with regard to FM are necessary to confirm our findings and the theory that CB1 can be used as a treatment target for FM.

## Data Availability

The data that support the findings of this study are available on request from the corresponding author. The data are not publicly available due to privacy or ethical restrictions.
